# Cytosolic phospholipase A_2_ regulates alcohol-mediated astrocyte inflammatory responses in HIV-associated neurocognitive disorders

**DOI:** 10.1038/cddiscovery.2015.45

**Published:** 2015-11-30

**Authors:** R Pandey, A Ghorpade

**Affiliations:** 1 Department of Cell Biology and Immunology, University of North Texas Health Science Center, Fort Worth, TX, USA

## Abstract

Alcohol (EtOH) abuse and HIV-1 infection remain leading public health problems not only in the United States but also across the world. Alcohol abusers have a significantly greater risk of HIV-1 infection than non-drinkers globally. In the United States, prevalence of EtOH abuse is over two-fold higher in HIV-1-positive individuals than that of the general population. Although alcohol abusers show neurodegeneration, exacerbated neuroinflammation and oxidative damage, the mechanism(s) by which EtOH regulates astrocyte inflammatory responses in HIV-associated neurocognitive disorders is unknown. Thus, we explored signaling pathway(s) involved in EtOH-mediated activation of human astrocytes with HIV-1 and subsequent alterations in their inflammatory functions. Alcohol exposure altered the morphology of astrocytes, proinflammatory responses and induced cytotoxicity in a dose-dependent manner. Time-dependent changes were also evaluated. EtOH and HIV-1 cotreatment decreased cell viability and proliferation, while increasing apoptosis and mitochondrial depolarization. EtOH and HIV-1 together increased the levels of proinflammatory molecules, interleukin-1*β*, tumor necrosis factor-*α*, CXCL8, tissue inhibitor of metalloproteinases-1 and more importantly, arachidonic acid, a known downstream target of cytosolic phospholipase A_2_ (cPLA_2_). Consistent with this observation, phospho-cPLA_2_ levels were augmented in HIV-1 and EtOH cotreatment as compared with HIV-1 or EtOH alone. Cyclooxygenase 2 was upregulated as measured by real-time PCR and western blot, whereas cotreatment of HIV-1 and EtOH decreased cytochrome P450-2E1 levels as compared with EtOH alone. Furthermore, we confirmed that blocking cPLA_2_ with arachidonyl tri floro methyl ketone, a cPLA_2_-specific inhibitor, effectively prevented cPLA_2_ phosphorylation and downstream outcomes. Thus, the present findings suggest that cPLA_2_ has a critical role in alcohol and HIV-induced astrocyte inflammation. In the future, cPLA_2_ inhibitors may present novel therapeutic tools to treat alcohol abuse and HIV-associated neurocognitive disorder comorbidity.

## Introduction

Alcohol (EtOH) abuse and HIV-1 infection remain significant public health problems. Drinkers have approximately 70–77% greater risk of HIV-1 infection than non-drinkers across the globe.^1,2^ The prevalence of alcohol abuse among HIV-1-positive individuals has been estimated as two to three times that of the general population in the United States (as reviewed in Justice *et al.* ^3^). As alcohol (EtOH) is water and fat soluble, it crosses cell membranes leading to altered immune responses and host resistance to infection.^4^ The brain is one of the major organs for EtOH action, and chronic intoxication results in significant modifications in brain structure and function, including neurodegeneration.^5–8^ During the course of HIV-1 central nervous system (CNS) infection, astrocytes respond to proinflammatory stimuli by releasing several cytokines and chemokines, which are key mediators of HIV-1-induced neuronal damage.^9,10^

Excessive production of inflammatory cytokines, chemokines and reactive oxygen species is a common molecular basis of pathogenesis in several neurodegenerative diseases such as HIV-1-associated dementia,^11^ Parkinson’s,^12^ and Alzheimer’s disease.^13^ In the post-antiretroviral therapy era, HIV-associated neurocognitive disorders (HAND), are associated with low levels of infection and activation of mononuclear phagocytes (MP). These MP potentiate neuroinflammation by activating neuroglia, ultimately disrupting neuronal function.^14^ Despite the availability of effective antiretroviral therapy, HAND continues to afflict between 15 to 69% of HIV-1-infected individuals.^15^

Alcoholism has long been associated with brain damage ranging from fetal alcohol syndrome to brain atrophy induced by years of chronic alcohol abuse. In rodents, chronic EtOH administration, increased microgliosis, enhanced inflammatory mediators, oxidative damage and reduced immune responses were evident before brain atrophy was prevalent.^8,16,17^ Therefore, neuroinflammation is a putative mechanism for EtOH-mediated neurodegeneration in alcoholics and is exacerbated during HAND.^17^ In previous studies, co-administration of EtOH and HIV-1 transactivator of transcription (Tat) upregulated proinflammatory cytokines expression in mouse brain.^18^ The direct toxic effects of HIV-1 proteins to neurons have also been extensively studied.^19,20^

Eicosanoids, including prostaglandins (PGE) and leukotrienes, have been implicated in the dysregulation of neuroinflammation during HAND and are increased during alcohol abuse.^21,22^ In astrocytes, PLA_2_ and cyclooxygenase (COX)-2 are upregulated during EtOH exposure, leading to increased PGE_2_ secretion.^22^ Of the four members of PLA_2_ enzymes family, only the cytosolic Ca^2+^-dependent PLA_2_ (cPLA_2_) is capable of catalyzing the hydrolytic release of arachidonic acid (AA) from membrane phospholipids.^23^ AA is an omega-6 (n-6) polyunsaturated fatty acid, esterified on the sn-2 acyl position of phospholipids (especially, phosphatidylethanolamine, phosphatidylcholine and phosphatidylinositol) in the membranes of mammalian cells.^24^ AA serves as the precursor for bioactive lipid mediators, the eicosanoids. In response to a wide variety of extracellular stimuli, AA can act directly as a lipid secondary messenger, be metabolized by cytochrome P450-2E1 (CYP2E1) to regulate oxidative stress,^25^ or get converted into eicosanoids by COX2, to regulate inflammation and immunity.^21,26^

Although the inflammatory and cytotoxic effects of EtOH and/or HIV-1 in animal models and human brain cells are well documented,^17–20^ the mechanisms through which EtOH and HIV-1 regulate astrocyte neuroinflammatory responses are unclear. In this study, we hypothesized that cPLA_2_ signaling may have an important role in EtOH-mediated astrocyte inflammation and cytotoxicity in the context of HAND. We demonstrate that EtOH and HIV-1 activate human astrocytes and together augment their activation, thereby further inducing neuroinflammation. We show that cPLA_2_ signaling may have a critical role in the regulation of astrocyte responses and targeting this pathway may lead to new therapeutic options for neurological impairment in HIV-1 infection and alcohol abuse.

## Results

### Alcohol induces a reactive phenotype in human astrocytes

**Figure 1:**
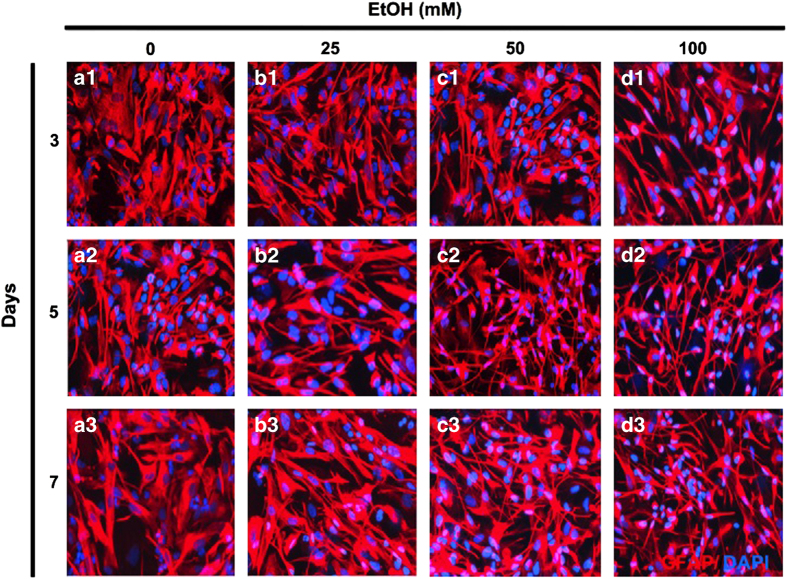
Alcohol (EtOH) induces a reactive phenotype in human astrocytes. Astrocytes were treated with 0 (**a1**–**a3**), 25 (**b1**–**b3**), 50 (**c1**–**c3**) and 100 (**d1**–**d3**) mM EtOH and incubated for 3 (**a1**–**d1**), 5 (**a2**–**d2**) or 7 (**a3**–**d3**) days. Fixed cells were immunostained with glial fibrillary acidic protein (GFAP, red) and counter-stained with DAPI (blue) to identify nuclei. Original magnification, ×200.

Blood alcohol concentration of 0.08% (22 mM EtOH) is the legal limit for driving in many states of the United States.^27^ Notably, 10 *µ*M-100 mM alcohol concentrations range from behaviorally sub-threshold to severe intoxication, respectively.^28^ Therefore, we used 25–100 mM EtOH concentration to explore whether EtOH altered astrocytes morphology in a dose- or time-dependent manner. Astrocytes were exposed to 0, 25, 50 and 100 mM EtOH for 3, 5 and 7 days. Astrocytes were fixed and immunostained for glial fibrillary acidic protein (GFAP) and 4′,6-diamidino-2-phenylindole (DAPI) to visualize cell body and nuclei, respectively (Figure 1). Upon treatment with increasing doses and durations of EtOH, astrocyte morphology changed to a reactive phenotype with constricted cell bodies, elongated processes and increased intensity of GFAP labeling; while control astrocytes retained bright GFAP staining distributed throughout cell body and processes. Interestingly, 50 mM EtOH on day 5 (Figure 1c2) was comparable to 100 mM EtOH on day 3 (Figure 1d1). However, by day 5, astrocytes showed comparable phenotypes at both 50 and 100 mM EtOH doses and remained so through day 7. Together, these data indicate that EtOH induces a reactive phenotype of astrocytes in a dose- and time-dependent manner.

### EtOH leads to astrocyte toxicity and regulates proinflammatory immune responses

**Figure 2:**
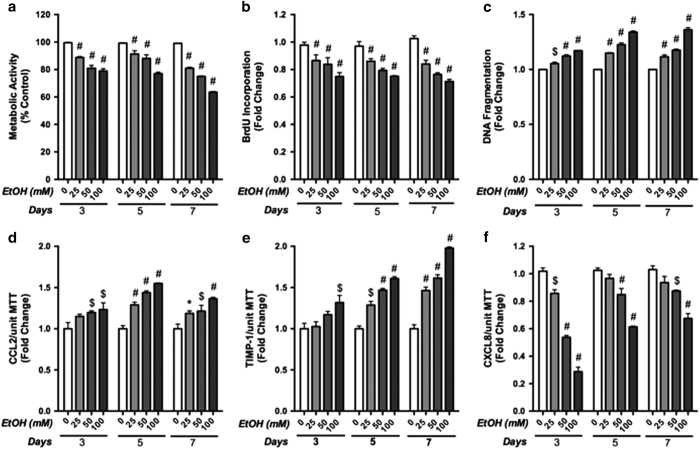
EtOH induces astrocyte toxicity and regulates proinflammatory responses. Metabolic activity (**a**), proliferation (**b**) and apoptosis (**c**) were assessed in astrocytes treated with 0, 25, 50 and 100 mM EtOH for 3, 5 and 7 days. In parallel experiments, cell supernatant levels of CCL2 (**d**), TIMP-1 (**e**) and CXCL8 (**f**) were measured by ELISA (ng/ml). Cumulative data from three independent astrocyte donors each analyzed in multiple replicates is shown as fold changes to the respective controls. Statistical analyses were performed using one-way ANOVA with Bonferroni *post hoc* test for multiple comparisons (*P<0.05, ^$^*P*<0.01 and ^#^*P*<0.001).

We next explored the effect of EtOH on astrocyte toxicity, proliferation and expression of proinflammatory mediators. Primary human astrocytes were exposed to 0, 25, 50 and 100 mM EtOH for 3, 5 and 7 days. Metabolic activity, proliferation and apoptosis were measured by MTT, BrdU incorporation assay and by DNA fragmentation (Figures 2a–c, respectively). Metabolic activity and cell proliferation significantly decreased, while apoptosis increased significantly upon treatment with EtOH in a dose-dependent manner (^#^*P*<0.001, respectively). As EtOH induced cytotoxicity and a reactive phenotype in astrocytes, we next assessed if the expression of proinflammatory mediators was also altered. In parallel experiments, supernatant levels of CCL2, TIMP-1 and CXCL8 were measured by ELISA. CCL2 and TIMP-1 levels increased in a dose-dependent manner, while CXCL8 levels decreased significantly as compared to controls (Figures 2d–f, *P<0.05, ^$^*P*<0.01, ^#^*P*<0.001, respectively). By 5 days, 50 mM EtOH significantly altered all parameters tested and thus was used to assess the combined effect of EtOH and HIV-1 in subsequent experiments. These data reveal that EtOH directly leads to astrocyte cytotoxicity and regulates astrocyte functions along with a reactive phenotype, proliferation and secretion of proinflammatory mediators.

### EtOH and HIV-1 comorbidity in astrocytes

**Figure 3:**
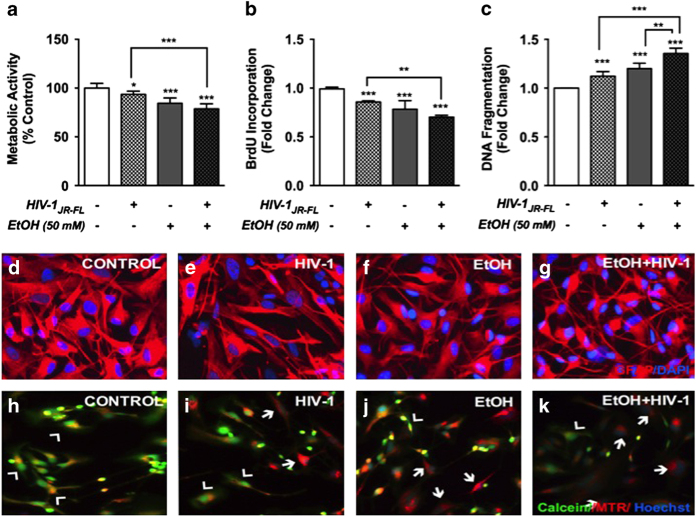
Effect of EtOH±HIV-1 on astrocyte toxicity, morphology and mitochondrial function. Astrocytes were exposed to EtOH (50 mM) for 5 days and treated with HIV-1 for 24 h. Metabolic activity (**a**), proliferation (**b**) and apoptosis (**c**) were assessed by MTT, BrdU incorporation and DNA fragmentation assays, respectively. All experiments were conducted using three independent astrocyte donors analyzed in multiple replicates and cumulative data normalized to controls are shown as fold changes. Statistical analyses were performed using one-way ANOVA with Bonferroni *post hoc* test for multiple comparisons (*P<0.05, **P<0.01 and ***P<0.001). In parallel, immunostaining was performed for glial fibrillary acidic protein (GFAP, red) and DAPI (nuclei, blue); (**d–g**; control (**d**), HIV-1 (**e**), EtOH (**f**) and EtOH and HIV-1 (**g**)). To monitor the mitochondrial permeability transition pore (mPTP) opening using calcein/CoCl_2_ (cobalt chloride) assay, astrocytes (control, **h**) were incubated with EtOH (50 mM, **j**) and HIV-1 (**i**) or in combination (**k**) for 24 h. Calcein (green) co-localized with Mitotracker (MTR, red) represents closed mPTP (arrowhead, yellow), while loss of green fluorescence represents opening of mPTP (arrow). Original magnification, ×200.

Next, to study the effect of EtOH on astrocyte responses during HIV-1 CNS infection, astrocytes were continuously treated with EtOH (50 mM, 5 days) and then with HIV-1_JR-FL_ (10 ng/ml HIV-1 p24, 24 h). Both metabolic activity and proliferation were significantly reduced by either HIV-1 or EtOH alone, while apoptosis levels were significantly increased as compared to controls (Figures 3a–c; *P<0.05, **P<0.01 ***P<0.001). Interestingly, cotreatment of HIV-1 and EtOH exacerbated these changes as compared to treatment with HIV-1 alone or untreated controls (Figures 3a–c; **P<0.01, **P<0.001, respectively). Immunostaining with GFAP (red) and DAPI (blue) revealed reactive astrocyte morphology upon treatment with HIV-1 or EtOH alone, which appeared more pronounced in cotreated astrocytes (Figures 3d–g). To test effects of EtOH and/or HIV-1 on mitochondrial function, astrocytes were treated with HIV-1_JR-FL_ (10 ng/ml HIV-1 p24) or EtOH (50 mM), alone and in combination, for 24 h and then the state of mitochondrial permeability transition pores (mPTP) was assessed by acetomethyl-calcein, cobalt chloride (CoCl_2_) quenching assay. Control astrocytes exhibited calcein (green) and mitotracker red (MTR) colocalization in mitochondria (yellow, Figure 3h, arrowhead), indicating closed mPTP. Calcein fluorescence decreased in astrocytes treated with HIV-1 or EtOH alone, indicating the opening of mPTP, a state that was augmented during cotreament (Figures 3i–k, arrows). Together, these data indicate that HIV-1 and EtOH cotreatment deleteriously affects astrocyte health by promoting mitochondrial pore opening that results in cell death and reduced proliferation.

**Figure 4:**
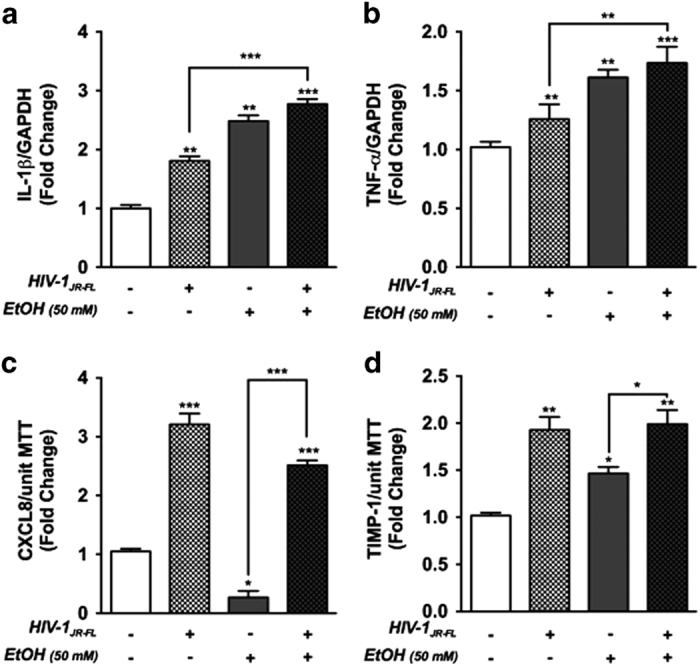
EtOH with or without HIV-1, alters astrocyte inflammatory responses. IL-1*β* (**a**) and TNF-*α* (**b**) mRNA levels were measured by real-time PCR in astrocytes exposed to EtOH±HIV-1 for 24 h. GAPDH was used as an internal normalizing control. In parallel experiments, astrocytes were exposed to EtOH for 5 days followed by HIV-1 for 24 h. CXCL8 (**c**) and TIMP-1 (**d**) protein levels in cell supernatant were analyzed by ELISA and then normalized to unit MTT activity. Cumulative data from three independent astrocyte donors, each assayed in multiple replicates are shown as fold changes to control. Statistical analyses were performed using one-way ANOVA with Bonferroni *post hoc* test for multiple comparisons (*P<0.05, **P<0.01 and ***P<0.001).

We next investigated changes in expression of proinflammatory mediators in response to EtOH and HIV-1 cotreatment. IL-1*β* and TNF-*α* mRNA levels were significantly increased in HIV-1 (**P<0.01), EtOH (**P<0.01) and cotreatment (***P<0.001 and **P<0.01 respectively) as compared to controls (Figures 4a and b). Interestingly, combined treatment of HIV-1 and EtOH significantly upregulated IL-1*β* and TNF-*α* mRNA expression in astrocytes as compared to HIV-1 alone (Figures 4a and b, ***P<0.001 and **P<0.01 respectively). Although CXCL8 levels increased significantly in astrocyte culture supernatants in response to HIV-1_JR-FL_ treatment (Figure 4c, ***P<0.001) and EtOH alone (50 mM, 5 days) significantly reduced CXCL8 level compared to control (*P<0.05). Interestingly, the increase in CXCL8 induced by HIV-1 treatment was reduced by 30% in the presence of EtOH; however, this decrease was not statistically significant. Similarly, TIMP-1 levels were significantly upregulated by either HIV-1 or EtOH alone compared to control (Figure 4d, **P<0.01 and *P<0.05, respectively); however, cotreatment was comparable to that of HIV-1 alone. Both CXCL8 and TIMP-1 supernatant levels were significantly greater in EtOH and HIV-1 cotreatment as compared with EtOH alone (Figures 4c and d, ***P<0.001 and **P<0.01, respectively). These results show that astrocyte proinflammatory responses are exacerbated during combined treatment of EtOH and HIV-1.

### EtOH and HIV-1 regulate cPLA_2_ signaling in human astrocytes

**Figure 5:**
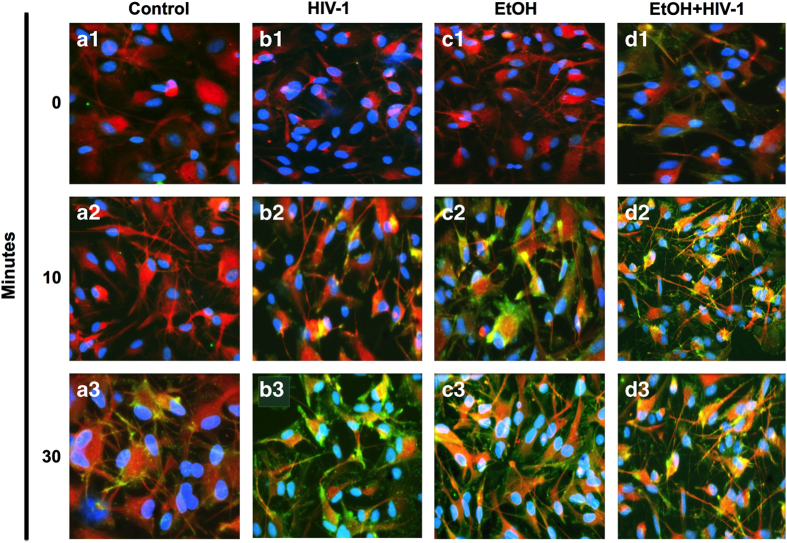
EtOH±HIV-1 induces cytosolic phospholipases A_2_ (cPLA_2_) phosphorylation in human astrocytes. Astrocytes were treated with 50 mM EtOH and/or HIV-1 for 0 (**a1–d1**), 10 (**a2–d2**) and 30 min (**a3–d3**). Cells were fixed and immunostained for glial fibrillary acidic protein (GFAP red), p-cPLA_2_ (green) and counter-stained with DAPI (blue) to identify nuclei. Increased levels of p-cPLA_2_ were observed within 30 min of EtOH and/or HIV-1 treatments. Original magnification, ×200.

**Figure 6:**
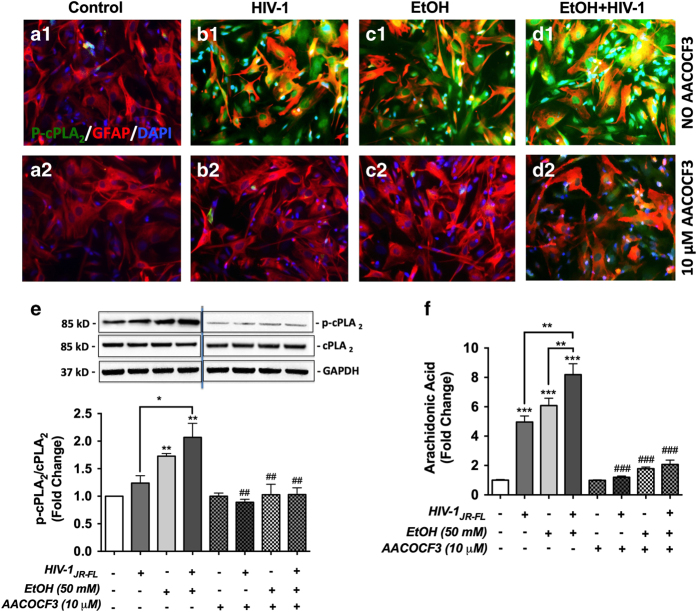
EtOH and/or HIV-1 alter astrocyte cPLA_2_ signaling. Astrocytes were pre-treated with AACOCF3 for an hour and then simultaneously treated with 50 mM EtOH and/or HIV-1 for 30 min. Cells were then fixed and immunostained with antibodies specific for phospho-cPLA_2_ and GFAP (green and red, respectively **a1–d2**). In parallel, equivalent amounts of total protein lysates were resolved by SDS-PAGE and immunoblotted for total cPLA_2_ and p-cPLA_2_. GAPDH was used as loading control. Representative immunoblot for p-cPLA_2_, total cPLA_2_ and GAPDH is presented, and cumulative densitometry data from three independent astrocyte donors is shown in the graph as fold changes to respective controls (**e**). Arachidonic acid levels were measured by ELISA and normalized to total protein. Three independent astrocyte donors were analyzed and cumulative data normalized to controls is shown as fold changes (**f**). Statistical analyses were performed using one-way ANOVA with Bonferroni *post hoc* test for multiple comparisons (*P<0.05, **P<0.01, ***P<0.001, ^##^*P*<0.01 and ^###^*P*<0.001). ^#^ Represents statistical significance for comparisons±AACOCF3, in respective treatments. Original magnification, ×200.

The first step was to confirm cPLA_2_ activation with HIV-1 and/or EtOH. Human astrocytes were treated with EtOH (50 mM) with and without HIV-1_JR-FL_ (10 ng/ml P24) for 0, 10 and 30 min. Astrocytes were fixed and immunostained for GFAP (red) and phospho-cPLA_2_ (p-cPLA_2,_ green) and counterstained with DAPI (blue) to identify nuclei. Phosphorylation of cPLA_2_ could be detected as early as 10 min with HIV-1, EtOH and combined treatments and p-cPLA_2_ was most evident in cotreated astrocytes, followed by EtOH and HIV-1 alone (Figures 5b2–d2). By 30 min, p-cPLA_2_ levels were increased in all conditions as compared to controls (Figures 5a3–d3). To confirm that EtOH and HIV-1 activate cPLA_2_ signaling, arachidonyl tri floro methyl ketone (AACOCF3), a cPLA_2_-specific inhibitor was employed. Human astrocytes were pre-incubated with 10 *μ*M AACOCF3 for 1 h, followed by simultaneous treatment with EtOH and/or HIV-1 for 30 min. Immunocytochemistry for p-cPLA_2_ demonstrated that EtOH and/or HIV-1 activate cPLA_2_, and AACOCF3 significantly blocked cPLA_2_ phosphorylation (Figures 6a1–d2). Subsequent experiments quantifying these changes via western blot showed that p-cPLA_2_ levels were significantly higher in EtOH alone and in cotreatments of HIV-1 and EtOH as compared to controls. However, HIV-1 and EtOH together increased p-cPLA2 significantly as compared to HIV-1 alone and the inhibitor successfully blocked these responses (Figure 6e, *P<0.05, **P<0.01, *P<0.05 and ^##^*P*<0.01). Total cPLA_2_ levels were unchanged across treatments. These findings confirmed that EtOH induces cPLA_2_ phosphorylation in astrocytes, which is augmented in the context of HIV-1.

We next examined downstream effects of cPLA_2_ activation. Intracellular AA levels, a known downstream product of cPLA_2_ hydrolytic activity was tested. 30 min post treatments of HIV-1 and/ or EtOH, total cell lysates were quantified by ELISA and confirmed that EtOH and/or HIV-1 significantly increased intracellular AA as compared to untreated controls. AA levels were significantly greater in HIV-1 and EtOH cotreatment as compared to either EtOH or HIV-1 alone (Figure 6f, ***P<0.001 and **P<0.01 respectively). Consistent with our hypothesis, cPLA_2_ blocking with AACOCF3 a cPLA2–selective inhibitor significantly inhibited p-cPLA_2_ and hydrolytic release of AA (Figure 6f). Among the phospholipase family, cPLA_2_ is the only enzyme capable of releasing AA from membrane phospholipids, these data confirm that cPLA_2_ is activated in astrocytes during combined treatment of EtOH and HIV-1.

**Figure 7:**
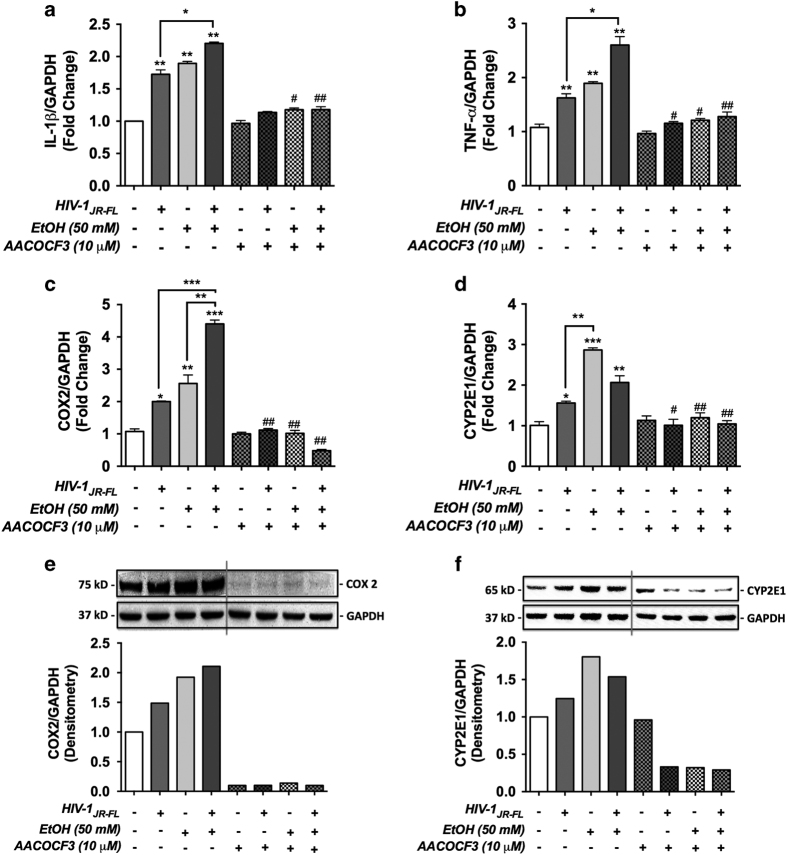
AACOCF3, a cPLA_2_-specific inhibitor reverses EtOH and/or HIV-1-mediated inflammatory responses. IL-1*β* (**a**), TNF-*α* (**b**), COX2 (**c**) and CYP2E1 (**d**), mRNA levels were measured in astrocytes exposed to AACOCF3 1 h, followed by continuous treatment with EtOH±HIV-1 for 24 h. In parallel experiments, whole-cell protein lysates were collected and resolved by SDS-PAGE, transferred and immunoblotted for COX2 and CYP2E1. GAPDH was used as normalizing control. Representative western blots with densitometry as fold changes to controls are shown (Figure 7e and f). Three independent astrocyte donors were analyzed in multiple replicates and cumulative data normalized to controls are shown as fold changes (**a**–**d**). Statistical analyses were performed using one-way ANOVA with Bonferroni *post hoc* test for multiple comparisons (*P<0.05, **P<0.01, ***P<0.001, ^#^*P*<0.05 and ^##^*P*<0.01). ^#^ Represents statistical significance for comparisons±AACOCF3, in respective treatments.

As COX2 and CYP2E1 are the AA metabolizing enzymes leading to the regulation of inflammation and oxidative stress,^23^ we next assessed IL-1*β*, TNF-*α*, COX2 and CYP2E1 levels in astrocytes, pre-treated with 10 *μ*M AACOCF3 for 1 h followed by activation with EtOH (50 mM) and HIV-1_JR-FL_, alone and in combination, for 24 h. Consistent with the pcPLA_2_ activation and downstream AA level changes, IL-1*β*, TNF-*α*, COX2 and CYP2E1 mRNA levels were significantly higher in all treatments compared with controls. Combined treatment of EtOH and HIV-1 augmented IL-1*β*, TNF-*α* and COX2 as compared with HIV-1 alone (Figures 7a–c, *P<0.05 and ***P<0.001 respectively), whereas HIV-1 and EtOH together showed robust elevation in COX2 levels as compared to HIV-1 and EtOH alone. COX2 levels, as measured by western blot also demonstrated similar responses (Figure 7e). CYP2E1 mRNA and protein levels, however, were decreased in cotreatments of HIV-1 and EtOH as compared to HIV-1 and EtOH alone. (Figures 7d and f ***P<0.001, *P<0.05 and **P<0.01). In parallel, cPLA_2_ inhibition via AACOCF3 significantly blocked EtOH- and/or HIV-1-induced changes (Figure 7a–f). These data confirm the involvement of cPLA_2,_ the downstream AA metabolizing enzymes in altering astrocyte function during HIV-1 and EtOH cotreatment. Taken together, these data suggest that cPLA_2_ signaling is upstream of AA and COX2, and may regulate astrocyte inflammatory responses.

## Discussion

Alcohol abuse exhibits a strong correlation with HIV-1 disease progression.^29^ EtOH exposure increases susceptibility to HIV-1 infection and stimulates HIV-1 replication in infected cells (reviewed in Bagby *et al.* ^30^). EtOH also contributes to inflammatory neurological impairment in HIV-1 patients^31^ and the development of HIV/AIDS-associated encephalitis or dementia.^17,32^ Dysfunction of blood–brain barrier (BBB), a common feature of HIV-1 neurodegeneration,^33^ is present during inflammation and chronic EtOH exposure in animal studies.^34^ Taken together, alcohol abuse and HIV-1 infection of the CNS have significant mechanistic commonality that results in combined toxic effects including neuroinflammation, neuronal injury or cognitive dysfunction.^35^

Although the role of astrocytes in HAND is evolving rapidly, the exact mechanism(s) of EtOH-induced astrocyte neuroinflammation in HIV-1 is unclear. We thus sought to explore the role of EtOH in the HIV-1-mediated inflammatory milieu of human brain astrocytes. Astrocytes are more resistant than neurons to oxidative and neurotoxic stresses including exogenous insults; yet, any damage to astrocytes can dramatically affect neuronal function. EtOH-induced detrimental effects on astrocytes can thus lead to alteration in neuron–astroglia interactions leading to developmental brain defects.^36,37^ HIV-1-associated neurotoxicity also includes secretory products from chronically activated glial cells, oxidative stress and glutamate-mediated excitotoxicity,^38,39^ which are similar to mechanisms mediating EtOH-induced neuronal injury.^8^ In the present study, we investigated how EtOH regulates HIV-1-mediated astrocyte inflammatory responses and novel pathways of EtOH controlled these responses in context to HAND.

Astrocytes are among the first to respond to brain injury and have an important role in fine-tuning neuroinflammation. It is well-documented that astrocytes become activated with EtOH, HIV-1 or HAND-relevant stimuli such as IL-1*β* and TNF-*α*.^17,40^ Indeed, CNS damage is often accompanied by astrocytic hypertrophy, proliferation and altered gene expression in association with inflammation, a process commonly referred to as reactive astrogliosis.^40,41^ In this study, we established that EtOH is a potent activator of reactive phenotypes of astrocytes. However, we also found that EtOH led to significant toxicity and concomitant reduction in astrocyte proliferation. Early studies into AIDS pathogenesis reported that apoptosis of CD8+ and CD4+ T lymphocytes has a key role in disease progression and that apoptosis of neuronal elements may also lead to the neuronal cell loss noted in patients.^42–44^ Increased apoptosis of neurons, astrocytes and microvascular endothelial cells also occurred in the CNS of macaques infected with a neurovirulent strain of simian immunodeficiency virus.^45^ Consistent with these observations, we showed HIV-1-mediated apoptosis of astrocytes that was further enhanced by EtOH.

Mitochondria have a critical role in cell injury by regulating power stores and contributing to oxidative stress and cell death during disease. In hepatocytes from EtOH fed animals, TNF-*α*-associated cytotoxicity involves the early loss of mitochondrial function, as demonstrated by mitochondrial depolarization due to permeability transition pore opening. In addition, AA-induced toxicity in CYP2E1-overexpressing hepatocytes is associated with the convergence of lipid peroxidation, intracellular Ca^2+^ release and activation of cPLA_2_.^46^ In primary human astrocytes, we also found increased mitochondrial depolarization and apoptosis accompanied by concomitant induction of cPLA_2_/CYP2E1 and enhanced AA release. Taken together, these mechanisms can directly contribute to mitochondrial dysfunction and oxidative stress in astrocytes during HAND and alcohol abuse.

In the pre-ART era, neuroinflammation was often associated with HIV-1 encephalitis (HIVE), initiated by brain resident microglia and infiltrating macrophages, the histopathological correlate of HIV-1 associated dementia (HAD).^47,48^ However, it is likely amplified by astrocytes, one of the most abundant cell types in the brain,^47,49^ despite not strongly supporting productive virus replication themselves. Astrocytes are important responders and mediators of HAND pathogenesis.^49^ Chronic EtOH significantly modifies brain structure and function leading to neurodegeneration. Our study revealed that alcohol exposure to astrocytes regulates a number of proinflammatory mediators including CCL2, CXCL8, IL-1*β*, TNF-*α* and TIMP-1. In a mouse model, chronic EtOH significantly increased TNF-*α*, CCL2 and IL-1*β* in response to LPS in brain.^50^ In subsequent studies using rat brain slice cultures, these studies confirmed the induction of cytokines along with inducible nitric oxide synthase in response to EtOH by NF-*κ*B activation in a dose- and time-dependent manner.^51^

In our study, EtOH and HIV-1 cotreatment increased the levels of proinflammatory molecules: IL-1*β*, TNF-*α*, CXCL8 and TIMP-1. Astrocytes initiate multiple autocrine and paracrine inflammatory signaling loops, leading to elevated production of cytokines, chemokines and other inflammatory molecules. In turn, these factors can enhance immune cells entry across the BBB, thereby further propagating neuroinflammation. Perturbation of astrocyte functions, such as dysregulation of extracellular glutamate clearance, also contributes to HIV-1-associated neurotoxicity.^52,53^ HIV-1 infection and exposure of non-neuronal cells to HIV-1 proteins have been associated with increased levels of inflammatory cytokines and chemokines such as IL-1*β*, IL-6, MIP-1*β*, RANTES and CCL2.^44^ Here, EtOH exerts a differential pattern of chemokine regulation in astrocytes, as evidenced by increased CCL2 in contrast to decreased CXCL8. Increased TIMP-1 reflects an early attempt of repair response combating extracellular matrix damage, as suggested in previous studies.^54,55^

Our data showed that HIV-1 together with EtOH synergistically activates the cPLA_2_ signaling pathway. A previous study reported that LPS treatment led to cPLA_2_ and PGE2 through ERK1/2-dependent pathway in astrocytes.^56^ Moreover, various other stimuli, including alcohol alone,^22^ ceramide^57^ and bradykinin^58^ have been shown to stimulate cPLA_2_ and regulate inflammatory and oxidative responses by MEK/p42/p44 MAPK pathways. Floreani *et al.* demonstrated that tyrosine phosphorylation of TLR4-Src kinase complex at the cell membrane–triggered Src kinase signaling and mediated activation of cPLA_2_ and COX2 on EtOH exposure. Chronic EtOH administration upregulated iNOS, COX2 and cytokine levels (IL-1*β*, TNF*α* and IL-6) in the cerebral cortex, and TLR4 deficiency protected the mice against EtOH-mediated glial activation and induction of inflammatory mediators.^22^

We investigated whether EtOH- or HIV-1-mediated cPLA_2_ activation released AA, a known downstream product of cPLA_2_ activity. AA is converted into PGE2 and leukotriene by COX2 and CYP2E1. EtOH-induced CYP2E1 generates reactive oxygen species leading to oxidative stress.^59^ Present study established exposure of EtOH or HIV-1 results in increased CYP2E1 and COX2 levels. Together, EtOH and HIV-1 cotreatment likely exacerbates inflammatory responses by activating cPLA_2_ pathway in astrocytes. AACOCF3 is a potent inhibitor of cPLA_2_. NMR studies show that the carbon chain of AACOCF3 binds in a hydrophobic pocket and the carbonyl group of AACOCF3 forms a covalent bond with the serine 505 in the active site. AACOCF3 is a 500-fold more potent inhibitor of cytosolic (c) PLA_2_ as compared to its soluble form.^23^ In our study, application of AACOCF3 as the cPLA_2_ selective inhibitor diminished downstream inflammatory outcomes in human astrocytes.

**Figure 8:**
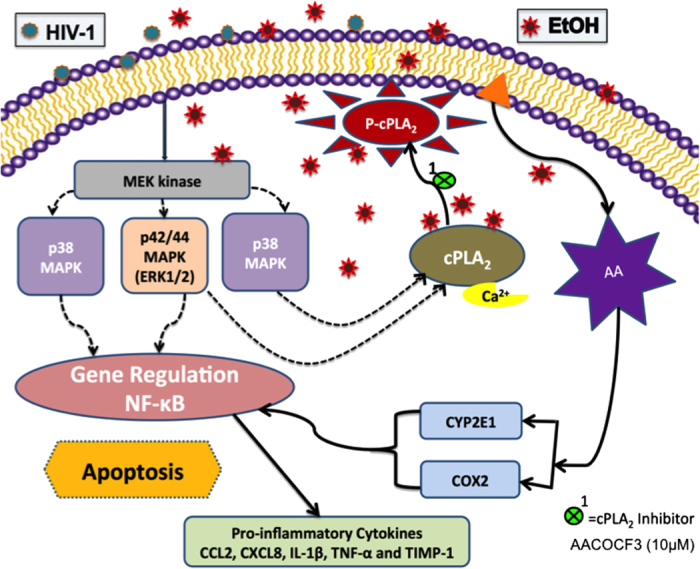
Role of EtOH-mediated cytotoxicity and neuroinflammation in context to HAND via cPLA_2_ signaling in human astrocytes. EtOH and HIV-1 alone or in combination induce cPLA_2_ phosphorylation through MAPK pathway. Phosphorylated cPLA_2_ releases arachidonic acid (AA) from phospholipids. AA is metabolized by COX2 and CYP2E1 enzymes into eicosanoids such as prostaglandin E2 (PGE2) leading to neuroinflammation via NF-*κ*B. We propose that cPLA_2_ signaling has a critical role in alcohol and HAND comorbidity.

To the best of our knowledge, this is the first report that HIV-1 together with EtOH, serve as potent co-modulators of astrocyte inflammatory responses with the key involvement of cPLA_2_, AA and COX2 activation. The salient feature of our study is cPLA_2_ signaling approach by which EtOH exposed astrocytes induced proinflammatory molecules leading to neuroinflammation in presence of HIV-1. Signaling mechanisms summarized in Figure 8 show that cytosolic PLA_2_ is an important pathway through which EtOH or HIV-1 and cotreatment increased AA, the downstream target of cPLA_2._ COX2 was also induced with EtOH, HIV-1 and combined treatments. Historically, COX2 inhibitors have been popular yet controversial therapeutic options. Our data suggest that in the context of HAND and alcohol abuse, targeting cPLA_2_, upstream of AA and COX2 will likely have greater impact in controlling inflammatory neurological outcomes.

## Materials and Methods

### Isolation and cultivation of primary human astrocytes

Human astrocytes were isolated from first and early second trimester aborted specimens as previously described.^60^ Briefly, tissues ranging from 80 to 120 days were procured in full compliance with the ethical guidelines of the National Institutes of Health, Universities of Washington and North Texas Health Science Center. Cell suspensions were centrifuged, washed, suspended in media and plated at a density of 20×10^6^ cells/150 cm^2^. The adherent astrocytes were treated with trypsin and cultured under similar conditions to enhance the purity of replicating astroglial cells. These astrocyte preparations were routinely >99% pure as measured by immunocytochemistry staining for GFAP).

### EtOH and HIV-1 exposure of astrocytes

Astrocytes were treated with alcohol (ethanol or EtOH; Pharmco, Brookfield, CT, USA) and HIV-1_JR-FL_ (p24, 10 ng/ml) alone and in combination at 37 °C and 5% CO_2_. HIV-1_JR-FL_ is a brain derived HIV-1 isolate and propagated *in vitro* as previously described.^61,62^ Astrocytes were incubated with 0, 25, 50 or 100 mM EtOH, and incubated for 3, 5 or 7 days at 37 °C. To maintain the appropriate EtOH concentration, EtOH was replenished every 24 h a reservoir with 2× concentrated EtOH was maintained in the incubator. The optimum EtOH concentration (50 mM) and EtOH exposure time (5 days) was selected for further experiments. After 5 days exposure of EtOH, cells were treated with HIV-1_JR-FL_ (p24, 10 ng/ml) for 24 h.

Astrocytes were pre-treated with or without 10 *μ*M Arachidonyl trifluoromethyl ketone (AACOCF3) for one hour followed by activation with HIV-1 and/or EtOH for concerned time points as standardized by other investigators.^22,23^

### Measures of cell viability, cytotoxity and proliferation

Cell viability was measured by MTT (3-(4,5-dimethylthiazol-2-yl)-2,5-diphenyl-tetrazolium bromide assay as described previously.^63^ Absorbance was measured using a microplate reader (Molecular Devices, Sunnyvale, CA, USA) at 490 nm.

Cell lysates were collected and DNA fragmentation was assessed by double-stranded DNA ELISA kit (Roche Diagnostics, Indianapolis, IN, USA) according to manufacturer’s protocol.

Cell proliferation was measured by BrdU (5-bromo-2′-deoxyuridine) incorporation assay (BrdU Cell Proliferation Assay Kit, Cell Signaling Technology, Danvers, MA, USA) as per manufacturer’s recommendations.

The calcein/Co^2+^-quenching technique was used to elucidate the mitochondrial permeability transition pore opening, leading to programmed cell death using the Image-iT LIVE Mitochondrial Transition Pore Assay Kit (Life Technologies Inc., Carlsbad, CA, USA) as previously described.^64^ Live cells were imaged using Nikon Eclipse TE-300 microscope with appropriate excitation and emission filters for fluorescein. In closed mPTP condition, the Co^2+^ quenched the green fluorescence of calcein only outside of mitochondria; therefore, colocalization of MTR reflected as green/yellow to mitochondria. After opening of mPTP, Co^2+^ quenches the calcein released from mitochondria, which shifts the color predominantly to red.

### Immunocytochemistry

Astrocytes were fixed in methanol:acetone (1 : 1) for 20 min at −20 °C. After blocking, the cells were incubated overnight at 4 °C with chicken or rabbit GFAP, p-cPLA_2_ antibodies (1 : 700, Dako, Carpinteria, CA, USA; 1 : 300, Abcam, Cambridge, MA, USA respectively). After washing three times in PBS, cells were labelled with anti chicken (594 nm, red) or rabbit (488 nm, green) Alexa Fluor secondary antibody (1 : 100, Life Technologies), for 1.5 h at RT. Following three PBS washes, cells were labeled with DAPI to visualize nuclei (1 : 800, blue, Life Technologies). All images were taken at ×200 magnification on Nikon Eclipse and processed by NIS-Element BR 3.2 software (Nikon Inc., Melville, NY, USA).

### RNA extraction and gene expression analyses

Astrocyte RNA was isolated as described elsewhere,^65^ 24 h post treatment and mRNA levels were assayed by real-time PCR. Taqman 5′ nuclease real-time PCR was performed using StepOnePlus detection system and inventoried gene expression assays (Life Technologies). Commercially available gene expression assays were used to measure IL-1*β* (Hs00174097_ml), TNF-*α* (Hs00174128_ml), CYP2E1 (Hs00559368_ml) and COX2 (Hs00153133_ml) mRNA levels. GAPDH (glyceraldehyde 3-phosphate dehydrogenase (4310884E)), a ubiquitously expressed housekeeping gene, was used as an internal normalizing control. The 25 *μ*l reactions were carried out at 48 °C for 30 min, 95 °C for 10 min, followed by 40 cycles of 95 °C for 15 s and 60 °C for 1 min in 96-well optical, real-time PCR plates. Transcripts were quantified by the comparative ΔΔCT method, and represented as fold change of control.

### Quantifications of proinflammatory mediators by ELISA

CCL2, TIMP-1 and CXCL8 protein levels (ng/ml) were determined using culture supernatants by sandwich ELISA (R&D Systems, Minneapolis, MN, USA) according to manufacturer’s protocol. Cell lysates were collected and intracellular levels of AA were assessed by ELISA kit (Bioassay Technology Laboratory, Shanghai, China) according to manufacturer’s protocol and normalized to total protein.

### Western blot analyses

Equal amounts of protein samples (20 *μ*g) were boiled with 4× NuPAGE loading sample buffer (Life Technologies) for 5–10 min, resolved by NuPAGE 4–12% Bis–tris gel and subsequently transferred to a nitrocellulose membrane using i-Blot (Life Technologies). The membrane was incubated with rabbit primary antibodies (cPLA_2_, 1 : 500, Abcam; p-cPLA_2_, 1 : 300, Abcam, CYP2E1, 1 : 500; Abcam and COX2, 1 : 500; Abcam) overnight at 4 °C, washed and then incubated with anti-mouse or anti-rabbit goat antibody IgG conjugated to horseradish peroxidase (1 : 1000, Bio-Rad) for 2 h at room temperature. The membrane was then developed with super-signal west femto substrate (Thermo, Rockford, IL, USA) in a Fluorochem HD2 Imager (proteinsimple, Inc., Santa Clara, CA, USA). GAPDH (1 : 5000, Cell Signaling) immunoblotting was used as a loading control.

### Statistical analyses

Statistical analyses were carried out using Prism 6.0 (GraphPad Software, La Jolla, CA, USA) with one-way analysis of variance (ANOVA) and Bonferroni *post hoc* test for multiple comparisons. Significance was set at *P*<0.05 and data represents means±S.E.M. Representative or cumulative data from minimum three independent astrocyte donors in multiple replicates is shown as fold changes to control.

## Abbreviations

AA: arachidonic acid
AACOCF3: arachidonyl tri floro methyl ketone
BBB: blood–brain barrier
Calcein-AM: acetomethoxy derivative of calcein
CCL2: C-C motif ligand 2
CNS: central nervous system
CoCl_2_: cobalt chloride
COX2: cyclooxygenase2
cPLA_2_: cytosolic phospholipase A_2_
CXCL8: C-X-C ligand 8
CYP2E1: cytochrome P450-2E1
DAPI: 4′,6-diamidino-2-phenylindole
EtOH: alcohol
GFAP: glial fibrillary acidic protein
HAND: HIV-1-associated neurocognitive disorders
IL-1*β*: interleukin-1*β*
mPTP: mitochondrial membrane permeability transition pore
MTT: (3-[4,5-dimethylthiazol-2-yl]-2,5-diphenyltetrazolium bromide)
PGE2: prostaglandin E2
TIMP-1: tissue inhibitor of metalloproteinases-1
TNF-*α*: tumor necrosis factor-*α.*
